# Developing Self-Assembled Starch Nanoparticles in Starch Nanocomposite Films

**DOI:** 10.1021/acsomega.2c05251

**Published:** 2022-12-02

**Authors:** Mahyar Fazeli, Juha Lipponen

**Affiliations:** Department of Bioproducts and Biosystems, School of Chemical Engineering, Aalto University, FI-00076 Aalto, Finland

## Abstract

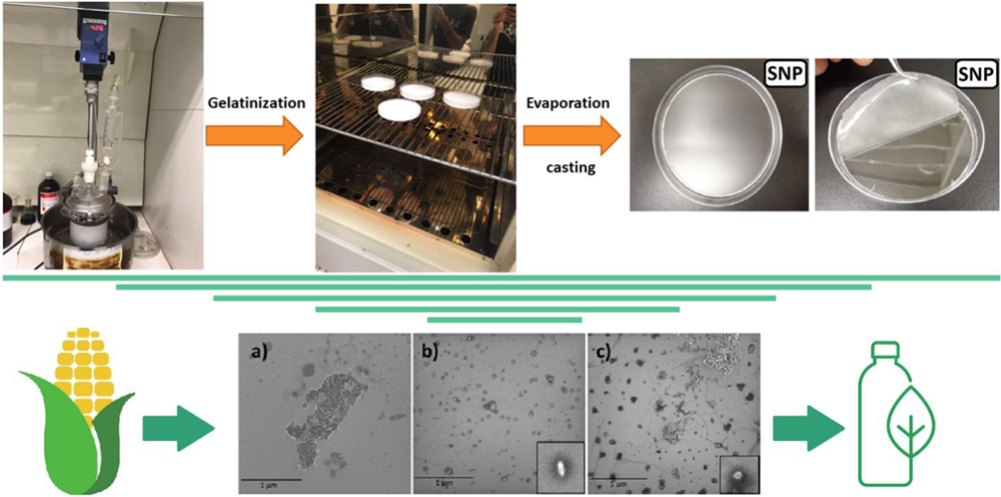

Starch nanoparticles (SNPs) are synthesized by different precipitation techniques using corn starch, and SNP films are prepared by the evaporation casting method. The morphological study is investigated by scanning electron microscopy (SEM) and atomic force microscopy (AFM). The distribution and sizes of precipitated SNPs after synthesizing are discovered by these methods as well. The crystallinity of the SNPs is studied by the X-ray diffractometry (XRD) method that demonstrates reduction compared to neat starch granules, and it is changed from A-style to V_H_-style after precipitation. The chemical bonding of different SNPs after the nanoprecipitation is analyzed by Fourier transform infrared spectroscopy (FT-IR). Thermogravimetric analysis (TGA) demonstrates the decomposition of starch nanoparticles and the starch matrix that is related to the depolymerization of carbon chains in the range of 260 to 350 °C. The mechanical properties of the SNP films versus the temperature changing are discovered by dynamic mechanical analysis (DMA). The water contact angles of SNP films are measured using a goniometer, and the results showed the hydrophobic surfaces of the prepared films. Our study indicates that SNPs have a promising impact on the properties of corn starch films, which would be useful in biodegradable packaging material.

## 1 Introduction

A significant advantage of nanoparticles (NPs) is their capability to be used in a broad range of applications in a variety of industries, including food, cosmetics, and healthcare. Natural starch is a nonallergic, plentiful polysaccharide that is renewable and biodegradable, making it an ideal candidate to be used in eco-friendly bioformulations.^1^ Starch is illustrated as a significant semicrystalline multistate structure that can create new nanoelements.^2^ Starch nanoparticles (SNPs) are frequently referred to as starch nanocrystals. It has been suggested that acid hydrolysis will produce starch nanocrystals when amorphous domains of semicrystalline granules are disrupted, while gelatinization may produce SNPs with amorphous matrixes.^3^ Other authors convey that it is almost impossible to distinguish between starch nanoparticles and starch nanocrystals since both phrases have been applied to the crystalline parts of starch after hydrolysis or other physicochemical treatments. They indicate that SNPs are used to represent elements with a nanoscale dimension.^4^

The preparation of SNPs can be categorized into two classifications based on the precursor material utilized for synthesis, bottom-up and top-down. Nanoparticles can be synthesized from a top-down process where larger volumes of materials or microparticles are broken down while nanoparticles can be generated by a bottom-up process made up of small primary cores made from building blocks of atoms or molecules that is controlled by thermodynamic standards such as self-assembly.^5^ In addition, a top-down process may be classified due to the number of stages it takes to prepare the final SNPs involved in simple or hybrid processes. Acid hydrolysis and enzymatic hydrolysis have been described as popular top-down simple methods for generating SNPs. In contrast to amorphous regions, crystalline regions are more resistant to acid hydrolysis after long periods of time and low yields of hydrolysis.^6^ An X-ray diffraction study was carried out by LeCorre et al. to see if the botany of starch affected crystallinity after acid hydrolysis. During this study, the amylose amount in starches was found to be the most crucial factor determining the crystallinity of SNPs, when starches of different botanical origins but with similar amylose contents were compared, there were no differences found.^7^

However, physical treatment, such as homogenization,^8^ ultrasonification,^9^ and extrusion,^10^ require less time and produce higher yields but can be difficult to control when it comes to crystallization. Combining enzymatic and acid hydrolysis for the preparation of SNPs has also been done with satisfactory results using the hybrid top-down method, since the procedure was able to be accomplished in a shorter time frame.^11^ Furthermore, hydrolysis has been combined with ultrasonication as a final refinement method in several studies. It is possible to reduce crystallinity for longer periods of time by ultrasonication, but this may change the starch’s X-ray diffraction pattern.^12^

Microemulsions and nanoprecipitation are the two most common methods of preparing SNPs from bottom-up processes. During nanoprecipitation, dilute polymer solutions are successively added to a solvent, which then results in the deposition of polymers at the surfaces after the removal of a semipolar solvent that is soluble with water. The microemulsion process includes the preparation of water-in-oil microemulsions containing aqueous parts dispersed in a steady oil phase reinstated by the interfacial film of the surfactant molecules functioning as nanoreactors where the synthesis of the preferred SNPs happens. Both approaches are gentle chemical techniques that are gaining increasing interest due to their effective control of shape, size, composition, and monodispersity of SNPs gained, instead of using toxic solvents or external energy sources.^13,14^

In this study, we attempted to produce starch films with different precipitation techniques of starch nanoparticles and analyze their physical properties such as morphology, crystallinity, and mechanical properties (stress/strain as a function of temperature). Furthermore, chemical and thermal properties of the prepared specimens are investigated by Fourier transform infrared spectroscopy (FT-IR) and thermogravimetric analysis (TGA), respectively. The outstanding results of hydrophobicity are obtained by using water contact angle measurements.

## 2 Materials and Methods

### 2.1 Synthasize of Starch Nanoparticles

First of all, 10.7 g of starch (Amido de Milho AMIDEX 3001) is added to the reactor containing 200 mL of distilled water for all types of prepared SNP films in this study. Then, 6.2 g of glycerol is added to all of the solutions except for SNP1. The solutions are kept mixing by the stirrer at a speed of 425 rpm. The temperature of the solutions is controlled by an oil bath which is located under the glass reactor, and the reactor is sunk into the oil for the heat exchange. The process of gelatinization starts when the temperature of the solution approaches 90 °C. After reaching 90 °C, the stirrer mixes the solution of SNP3 for 30 min and other samples for 60 min. Then, 200 mL of absolute ethanol are added dropwise to the gelatinized starch solutions except for SNP4 under active mechanical stirring. The gelatinized starch solutions are normally cooled down to room temperature. Afterward, 200 mL of absolute ethanol are added dropwise to all the solutions at room temperature. Thereafter, the solutions are kept under stirring for more than 5 min. Finally, 30 mL of each solution are put in a Petri dish, and they need to be on the furnace with a temperature of 50 °C for 24 h. The difference between the four types of the prepared solutions is summarized in Table 1.

**Table 1:** Preparation of Different SNP Films

film	content of glycerol	duration of gelatinization	addition of alcohol
SNP1	0	60 min	at 90 and 25 °C
SNP2	6.2 g	60 min	at 90 and 25 °C
SNP3	6.2 g	30 min	at 90 and 25 °C
SNP4	6.2 g	60 min	just at 25 °C

Furthermore, the mean size, size distribution, and relative standard deviation (RSD) of the SNPs are calculated through a dynamic light scattering (DLS; Malvern, Zetasizer NanoZS, U.K.). Two milliliters of obtained solutions are added to cuvettes and measured.

### 2.2 Scanning Electron Microscopy (SEM)

A scanning electron microscope (Vega3 Tescan Co. Ltd., Brno, Czech Republic) with an acceleration voltage of 15 kV is used to consider the microstructure of the starch nanoparticles on the surface of the samples. All of the analyzed samples are coated by gold with 3 nm as the thickness of the coating using a LEICA MICROSYSTEMS EMQSG100, USA. Furthermore, the morphology and the size of the nanoparticles are analyzed by scanning electron microscopy (Helios Nanolab, FEI). For the analysis, one droplet of the film solution is deposited on a (110) oriented silicon wafer (1 × 1 mm) and dried on a heating plate at 50 °C. This procedure is done a total of four times. The samples are fixed on a stub by a carbon tape. The nanoparticles size is calculated using a digital imaging analysis program.^15^

### 2.3 Atomic Force Microscopy (AFM) of SNP Films

An atomic force microscope (1 M Plus, JPK Instruments, Germany) is used to image the samples. The surface topography of the prepared films is obtained to exhibit the nanostructure of the starch nanoparticles on the surface. Furthermore, the dimension of the particles is measured by using ImageJ software. Images are obtained in dynamic mode using a Micromasch NSC 14/AIBS cantilever with a nominal spring constant of 5 N/m.^16^

### 2.4 Dynamic Mechanical Analysis (DMA) of SNP Films

Dynamic mechanical analyses are accomplished in a TA Instrument (Q800 DMA) with prepared SNP film samples. The dimension of the specimen is approximately 35 mm × 10 mm × 2 mm. The analysis is carried out at room temperature and 1 Hz in 0.01 N force-controlled deformation mode, from −80 to 150 °C, at a heating rate of 3 °C/min. The samples are kept at 21 °C and 50% relative humidity before the test for a period of 48 h.^17^

### 2.5 Fourier Transform Infrared Spectroscopy (FT-IR) of SNP Films

FTIR spectra are recorded to study the effect of the different conditions of nanoparticle formation and the functional groups created on the SNP films using a TA Instruments SDT-Q600 spectrometer, with a resolution of 5 cm^–1^ in the range of 800–4000 cm^–1^ using ATR mode. The chemical structure of starch nanoparticles on the samples might be proven by this method based on the formed peaks corresponding with specific chemical bonds.^18^

### 2.6 Crystalline Properties of SNP Films

The crystallinity of SNP films is gained by a Bruker D8 DISCOVER (Germany) diffractometer in the diffraction angles range 2θ = 10° and 40°, using a wavelength of 1.540A° with Cu Kα radiation and 90 s of time per step and a step size of 0.025°. The samples are placed in the sample holder, and the experiment is achieved in a static mode.^19^

### 2.7 Thermogravimetric Analysis (TGA) of SNP Films

Thermogravimetric analysis is accomplished to appraise the degradation characteristics of the SNP films. The thermal stability of three specimens for each kind of samples is distinguished by using a thermogravimetric analyzer (THERMO NICOLET 6700 spectrometer), with the nitrogen flow rate of 20 mL/min as the atmosphere and a heating rate of 10 °C/min. The weight of each sample is 15 mg, and they are subjected to a temperature range of 25 to 600 °C.^20^

### 2.8 Water Contact Angle of SNP Films

The surface contact angle of the prepared samples is measured by optical contact angle goniometer (NRL A-100-0; Ramé-Hart, NJ) to investigate the hydrophobicity of the obtained films. A three-axis horizontal tilt stage is utilized to make the surface of the samples perpendicular to the pipet. A droplet of water of about 10 μL is pipetted on the center of the surface at 25 °C. The water contact angles outcome of each sample is listed immediately and 100 s after the release when the droplet status stabilized.^21^

## 3 Results and Discussions

### 3.1 Scanning Electron Microscopy (SEM) of SNP Films

**Figure 1:**
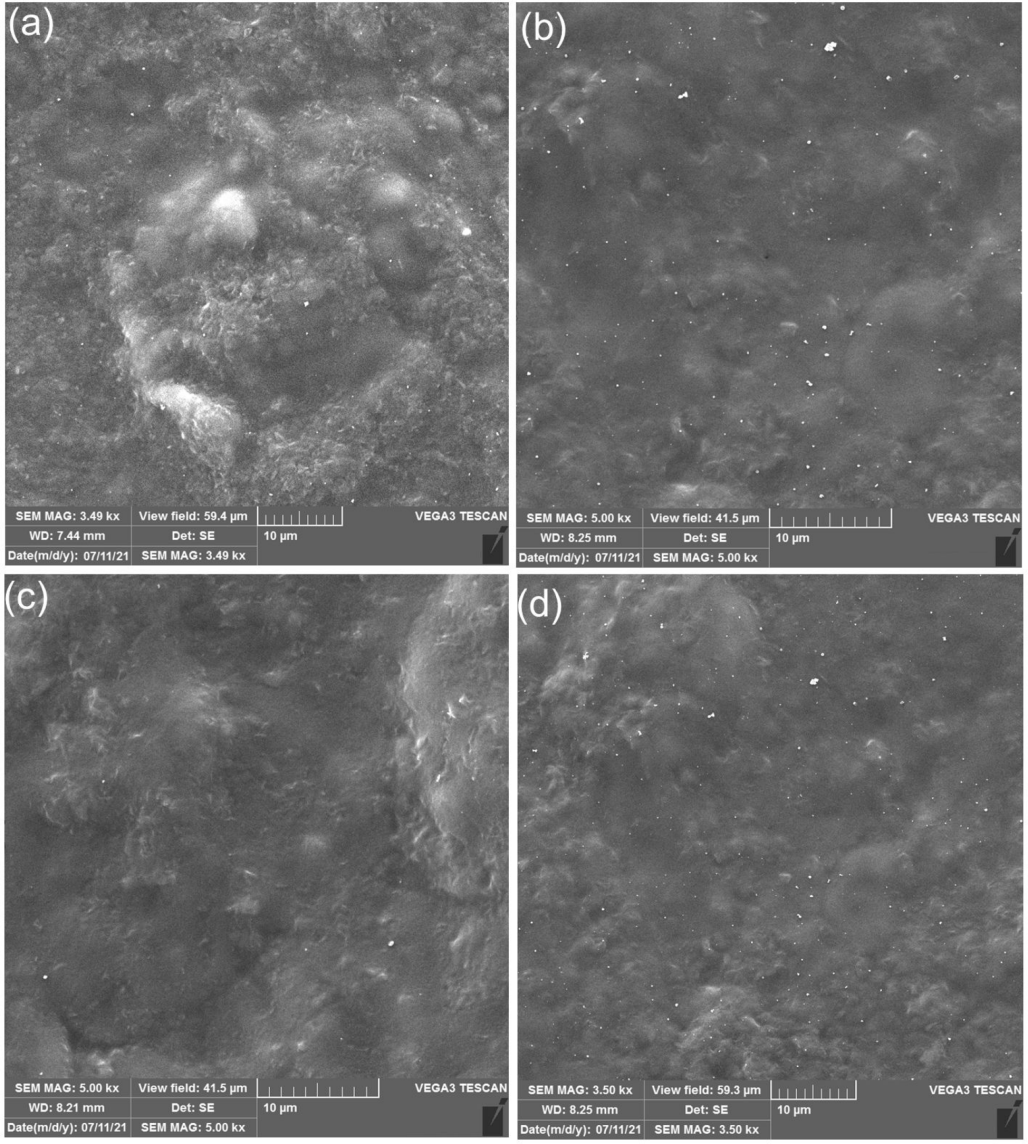
Scanning electron microscopy (SEM) of SNP1 (a), SNP2 (b), SNP3 (c), and SNP4 (d).

During the process of SNPs precipitation, native granular corn starch was gelatinized in water and formed a starch paste. The preparation of SNP in water by the dropwise addition of ethanol results in the nanoparticles (Figure 1a). Most of the SNPs possess sizes in the range from 50 to 300 nm. It is discovered that the stability of the nanoparticle suspensions is an important prerequisite for preparing these novel nanoparticles. The interaction of the hydrogen bond between starch nanoparticles and starch paste plays an important role in the stability of the precipitated nanoparticles in the suspension. The interaction of nanoparticles and ethanol seems to decrease the aggregation of nanoparticles, and large particles of starch reduce in Figure 1c. As shown in Figure 1d, no residual granular structure of starch is observed in the continuous phase. At a high temperature, water and glycerol are known to physically break up the granules of corn starch and disrupt intermolecular and intramolecular hydrogen bonds and make the native starch plastic. The distribution of the nanoparticles in the polymer matrix is shown in Figure 1b. Starch nanoparticles are dispersed well in the film matrix and without obvious aggregation, which is attributed to the strong interaction because of chemical similarities between ethanol and cornstarch in the film matrix. The number of nanoparticles for the SNP3 sample decreased in comparison to the other prepared films (Figure 1a). The reason might be the duration of gelatinization for the SNP3 sample, which is 30 min. As can be seen, more nanoparticles can be found on the surface of SNP2 because of the long duration of gelatinization, glycerol content, and addition of ethanol two times in the process.

**Figure 2:**
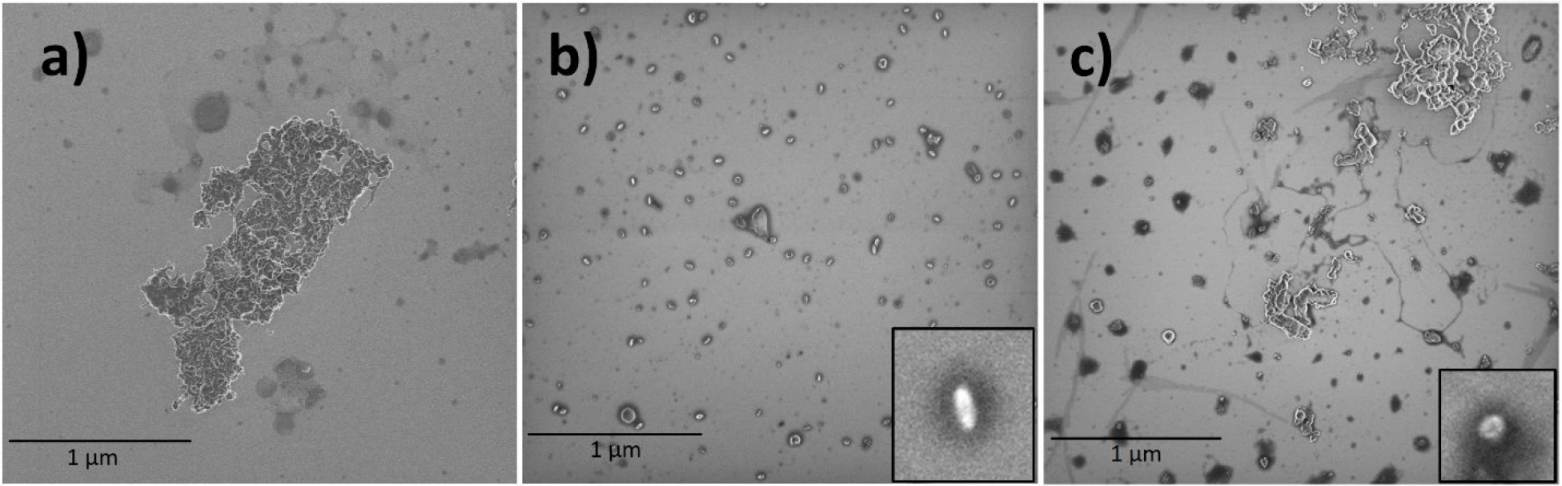
Scanning electron microscopy (SEM) of the nanoparticles’ throw nanoprecipitation at 90 and 25 °C (a), 25 °C (b), and 90 °C (c).

A nonsolvent must be added to the polymeric solution in order to obtain starch nanoparticles. Nanoparticle precipitation is the result of the diffusion between a nonsolvent and solvent caused by the addition of a nonsolvent. In accordance with the parameters, the particle shapes and sizes can differ. The nanoparticles formed when ethanol was added to the hot gelatinized starch and cooled down again to room temperature (Figure 2.a). In comparison to other methods of formation, they tend to be more agglomerated and larger in size. This is due to the strong hydrogen bonds between nanoparticles that result from the diffusion of the solvent and nonsolvent.

On the other hand, the starch molecular agitation is low while the nonsolvent is added to the gelatinized starch only when it is already cooled down to room temperature. Therefore, smaller sizes of particles will be formed, and less agglomeration will occur as illustrated in Figure 2b. On the contrary, the molecular agglomeration and particle size of starch will be greater when the nonsolvent is added to the hot solution, as indicated in Figure 2c.

### 3.2 Topography of SNP Films

**Figure 3:**
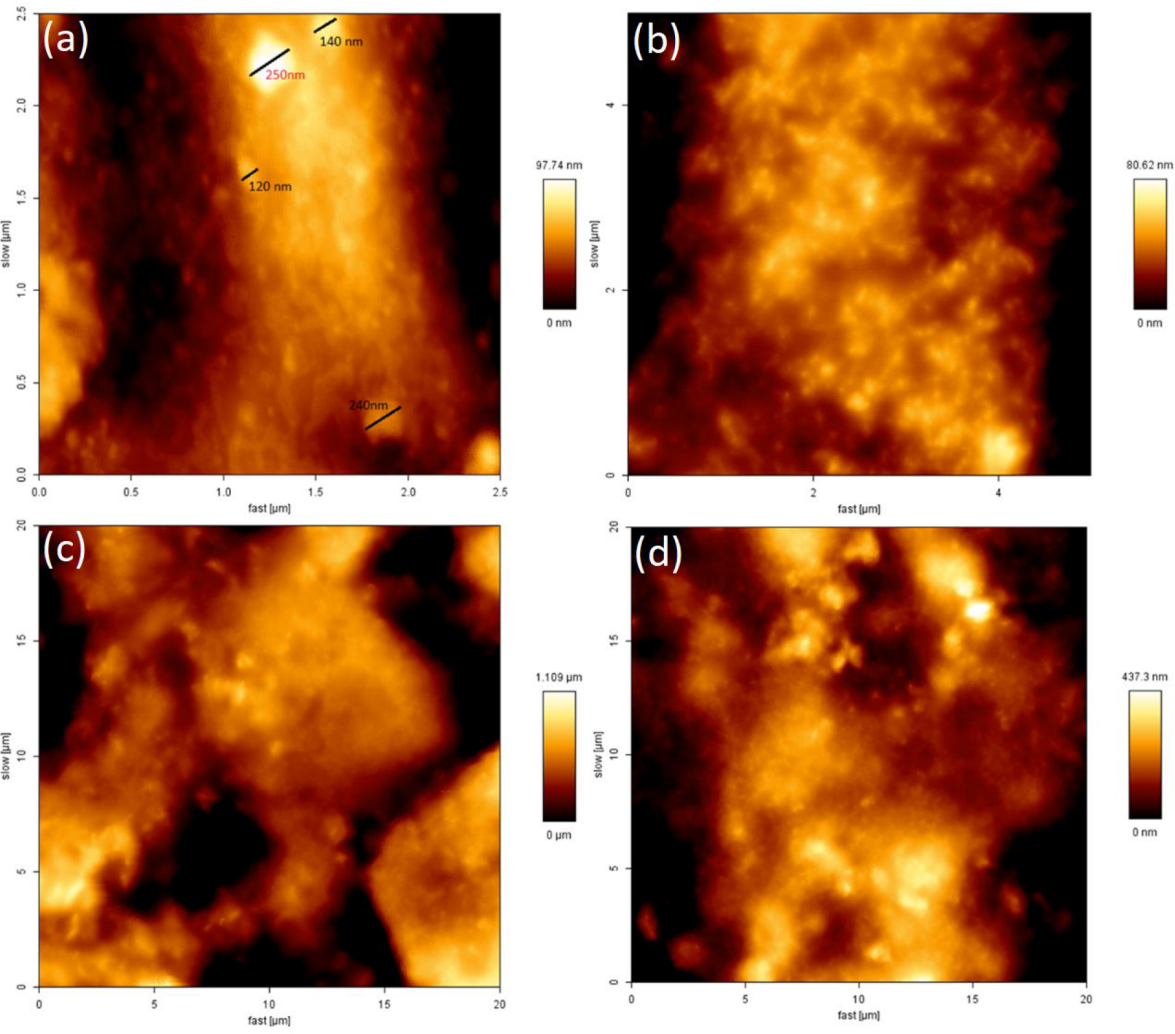
Atomic force microscopy (AFM) of SNP1 (a), SNP2 (b), SNP3 (c), and SNP4 (d).

The qualitative (morphology) and quantitative (roughness) parameters of the films are analyzed by AFM. It is used to examine the dispersion of SNPs in the starch matrix. As can be seen in Figure 3, those zones with the dark area are concavities enriched with starch, while those with brighter areas are protrusions enriched with starch nanoparticles. There is a relatively sharp contrast between starch and SNP phases. The control film presented a uniform and smoother surface across the area. Due to the incorporation of SNPs, surface roughness increased, and the uniformity of nanocomposite films decreased. This is in agreement with the findings of Díaz-Visurraga et al.^22^ and Zhou et al.^23^ Díaz-Visurraga et al.^22^ have stated that the random dispersion of starch nanoparticles in the starch film matrix led to enhancement of the film surface roughness. Furthermore, based on the results of the present study, it seems that the low hydrophilicity of starch in comparison to the film components could be another probable reason for the tendency of starch nanoparticles in the external part of the starch film, giving rise to the surface roughness.

The mean size, RSD, and particle size distribution of the SNPs are presented in Table 2. Although different samples have diverse size distributions, all of the SNPs exhibit a single peak, demonstrating that the solutions have a uniform particle size distribution. Among all of the prepared solutions, SNP1 has the largest particle size because of the lack of glycerol and agglomeration of the starch granules. On the other hand, SNP2 contains the finest nanoparticle because of longer time of gelatinization.

**Table 2:**
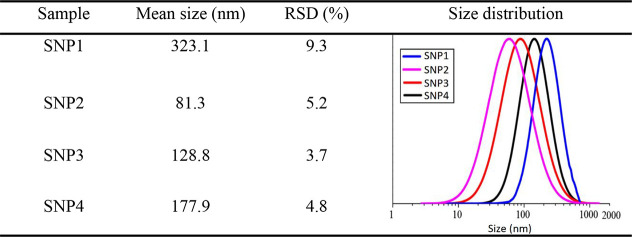
Statistics of Nanoparticles Size

### 3.3 Dynamic Mechanical Analysis (DMA) of SNP Films

**Figure 4:**
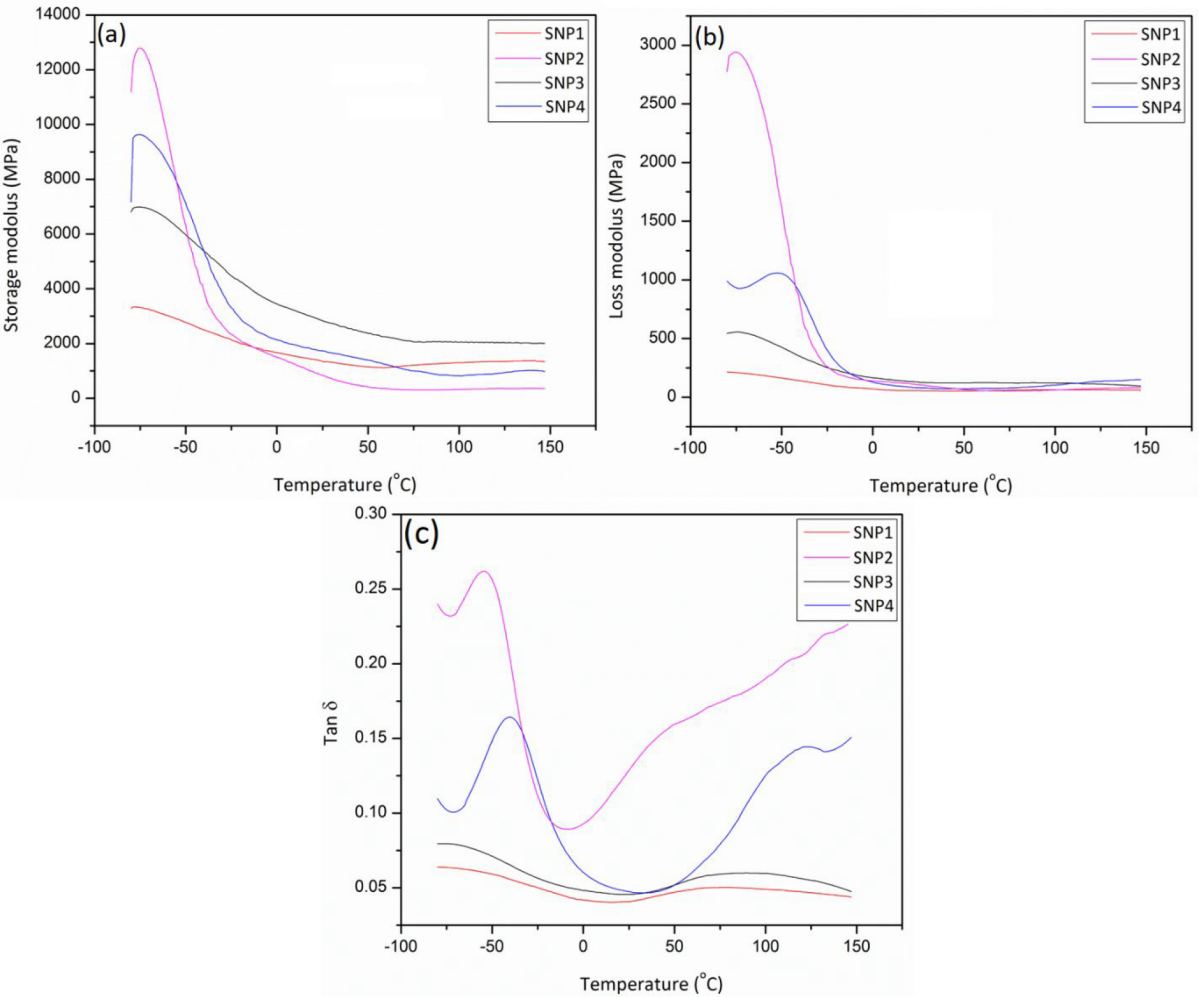
Storage modulus (a), loss modulus (b), and loss factor (tan δ) (c)/temperature curves of SNP films.

The purpose of this experiment is to understand the mechanical performance of polymeric films when they are subjected to various temperatures instead of room temperature.^24,25^ According to Figure 4, the storage modulus, the loss modulus, and tan δ of fabricated films are illustrated as a function of temperature. In practice, polymeric films exhibit distinct properties at different temperature ranges. Figure 4a illustrates the storage modulus of various SNP films as a function of temperature at a frequency of 1 Hz. Clearly, SNP1 confirms the minimum storage modulus quantity, since it gives a small degree of stiffness. Under the examined temperature range, all SNPs that do not contain glycerol as a plasticizer show significant improvements in storage modulus compared to SNP1. Starch nanoparticles have remarkably high stiffness properties, allowing them to dramatically constrain the movement of polymeric chains made of thermoplastic starch. It contributes to improvements in film stiffness of all SNP films.^26^ The loss modulus is calculated based on the energy loss during heat or cycle deformation and is regarded as a viscous reproduction of the material. Having a variety of starch nanoparticles in prepared films, the loss modulus exhibits comparable trends with the storage modulus, as demonstrated in Figure 4b. Increasing the glycerol content and duration of gelatinization improved the loss modulus of the films across the entire temperature range examined. As compared to all specimens, SNP2 film exhibited the highest loss modulus. The synergic improvements in loss modulus for SNP2 demonstrate homogeneous dispersion and great physical interaction between starch nanoparticles and the thermoplastic starch matrix. It is possible that the results are due to the evaporation of water and ethanol, as well as the development of adequate starch nanoparticles within the films. As described previously, the nanoparticles reduced the movement of the molecular chains of the TPS, thereby enhancing its thermal stability.^27^

Figure 4c illustrates the temperature change versus the damping factor change (represented by tan δ) for different prepared samples. The apex of tan δ is linked to the internal energy loss of a film, and it represents a material’s viscoelastic behavior. Since the nanoparticle concentration enhances the damping factor of the TPS, the plasticized SNP films expose a relatively higher tan δ apex. An increase in the tan δ outcome demonstrates that the films will become more viscous after raising the temperature. The SNP films demonstrated improved dynamic mechanical properties when compared to neat TPS films.^28^ A positive displacement in the tan δ apex for the SNP film might be the result of the strong chemical interaction between the TPS matrix and the nanoparticles, which limited the mobility of the polymer chains in the proximity of the nanoparticles.

### 3.4 Fourier Transform Infrared Spectroscopy (FT-IR) of SNP Films

**Figure 5:**
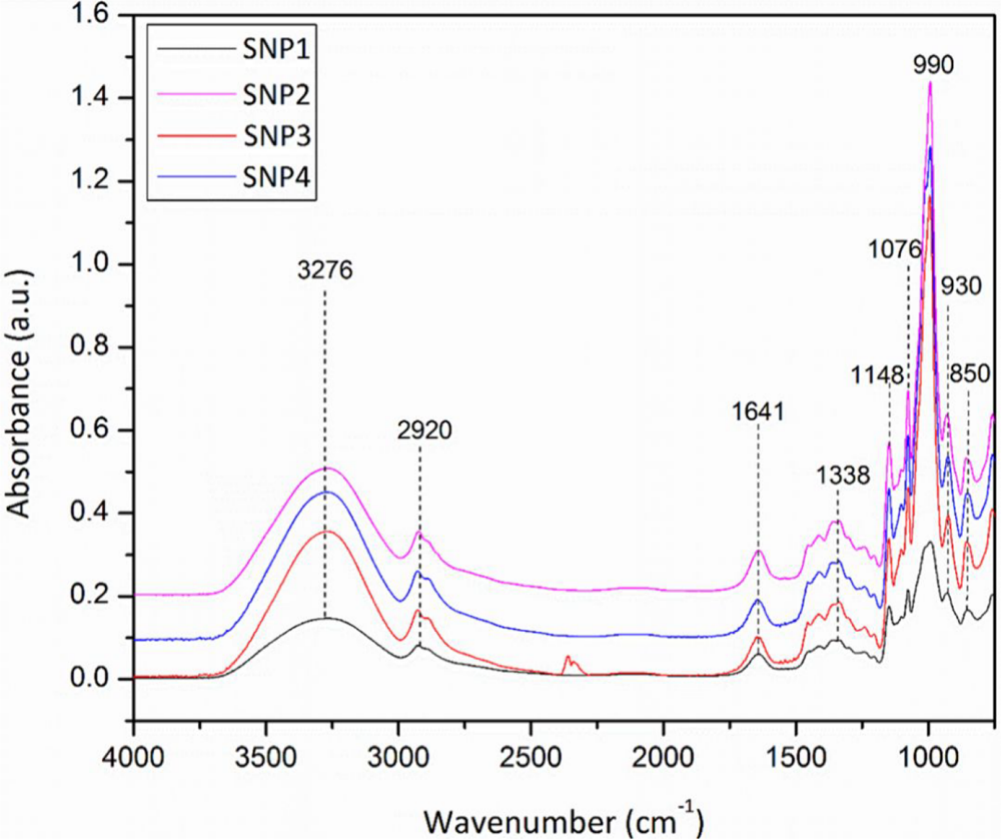
FT-IR curves of SNP films.

The existence of the different elements in the provided specimens may be estimated by checking the translocations of the absorption infrared peaks related to the wavenumber along with the interactions among the functional groups. Figure 5 exhibited the FTIR spectra of the SNP films. The characteristic peak happened at 1641 cm^–1^, which is considered to be the firmly bound structural water existing in the starch, associated with the angular deformation of hydroxyl groups. The absorption peaks in the range of 1000 and 1200 cm^–1^ are representative of the −C–O– stretching on the polysaccharide structure. The peak at 2920 cm^–1^ is attributed to the asymmetric C–H stretching. The spectra display a resemblance to the investigation explained by Akrami et al.,^29^ which represents a wide peak in the range of 3000 to 4000 cm^–1^ attributed to the vibration of the O–H functional groups’ deformation. The peak 1338 cm^–1^ relates to the angular distortion of the C–H bond. The set of peaks in the range of 900 to 1200 cm^–1^ is assigned to the stretching C–O–C glycosidic bonds.

Furthermore, accretion in the intensity of the peaks by the addition of glycerol is perceived veritably, which is associated with the glycerol interactions and the matrix of starch. The glycerol as the plasticizer is a low molecular weight compound and gets into the spiral structure of starch by destroying the intermolecular bonds. The principal variations of the peaks among plasticized starch matrixes with disparate glycerol contents are in the 3276 cm^–1^ peak, demonstrating alterations in the hydrogen bond model of the starch and glycerol combination. The absorption bands at 3276 cm^–1^ are allocated to O–H stretching vibrations associated with free and inter- and intramolecularly bound hydroxyl groups. In comparison with the SNP1 film, an increase in intensity observed for the films obtained after SNP formation indicates an increase in intermolecular hydrogen bonding mostly because of glycerol addition between the matrix and starch nanoparticles.^30^ On the other hand, the intensity of the peak at 990 cm^–1^ is different among the prepared samples, associated with the angular deformation of the C–O–C glycosidic bonds.

### 3.5 Crystalline Properties of SNP Films

**Figure 6:**
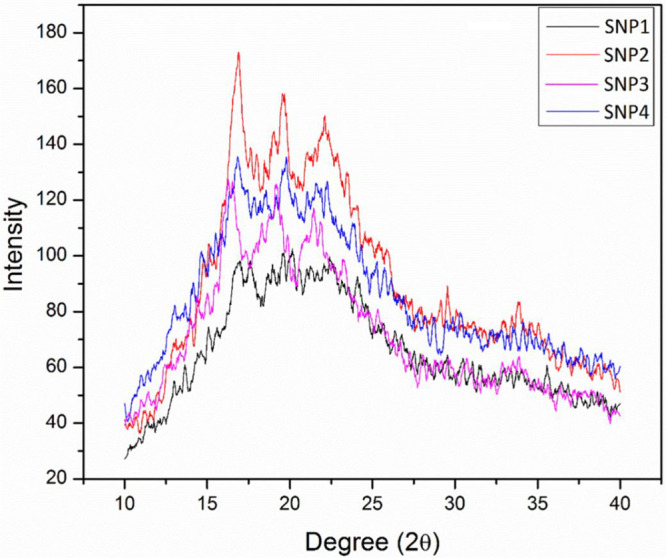
X-ray diffraction of SNP films.

Figure 6 illustrates the XRD diffraction pattern of the prepared films. As demonstrated before, native corn starch possessed the A-style crystallinity. In SNP, the V_H_-style crystallinity, which is different from A-style crystallinity in corn starch, might have originated from a unique helical formation (inclusion complex) fabricated by amylose and glycerol as the plasticizer. The plasticized corn starch also exhibits a similar V_H_-style crystallinity in its structure. During delivering the ethanol into the starch solution, the gelatinized SNPs are precipitated slowly. Consequently, the gelatinization annihilates A-style crystallinity of the starch, and starch nanoparticles demonstrate the V_H_-style crystallinity.

As can be seen in Figure 6, the crystallinity of the SNPs dramatically decreased in comparison with neat starch granules. These outcomes propose that the homogenization probably influenced the form of the double-helical chains of the starch in the SNPs as XRD examination may identify the long-chain crystalline structure of the granules like the packing order of double helices.

### 3.6 Thermogravimetric Analysis (TGA) of SNP Films

**Figure 7:**
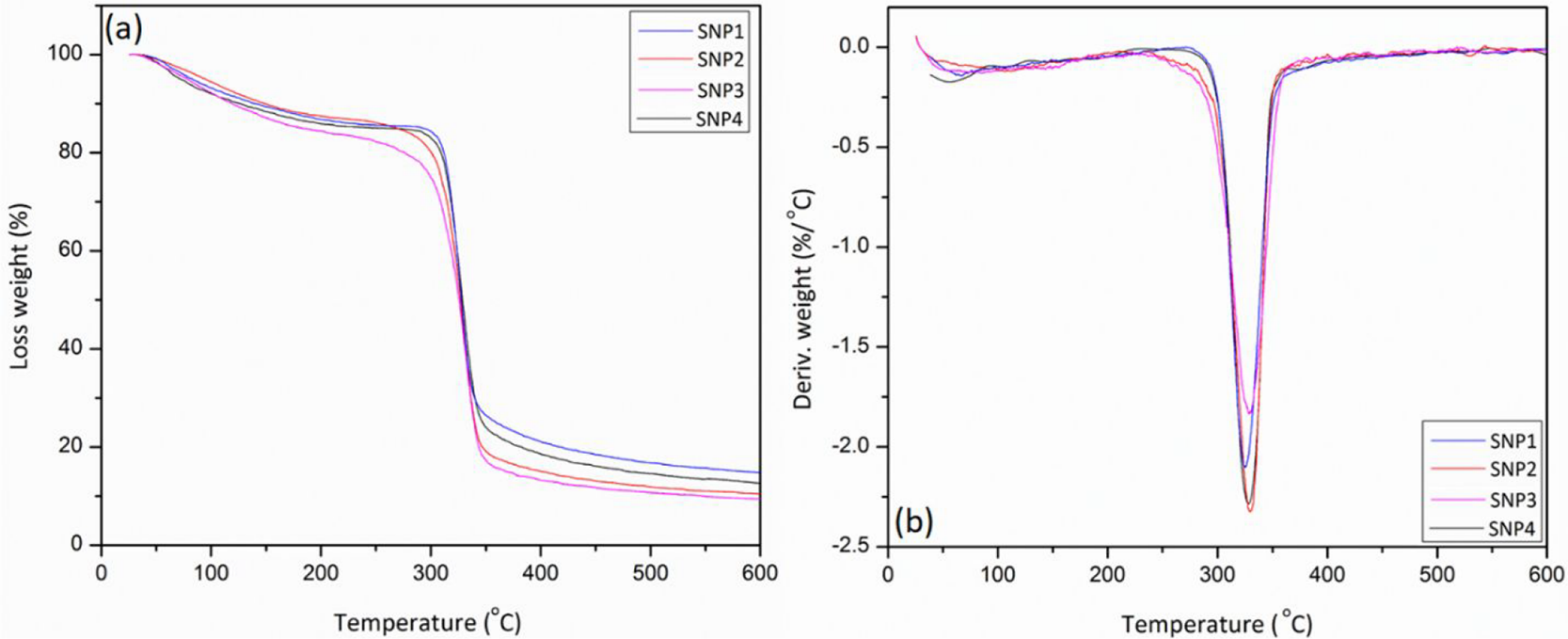
Thermogravimetric analysis (TGA; a) and derivative thermogravimetric (DTG; b) curves of SNP films.

Figure 7a demonstrates thermogravimetric analysis curves of the SNP films. Also, Figure 7b shows the first derivative thermogravimetric (DTG) curves of the same samples as well. The thermogravimetric analysis curves of the SNP films exhibit three degradation stages. The primary step of the degradation is attributed to the dehydration (the loss of structural humidity by evaporation) and low molecular weight compounds between 50 and 120 °C. The second and the main degradation steps are demonstrated between 260 and 350 °C, which is associated with the glycerol-rich phase of the film. The mass-loss rate for this stage is the fastest one among all of the steps, which is attributed to the decomposition of starch nanoparticles and the starch matrix related to the depolymerization of carbon chains and omission of −OH groups. The final stage occurred between 350 and 600 °C which is due to the common carbonization of the nanocomposites because of the starch-rich phase of the matrix. The starch nanoparticles increased thermal stability, as is seen in Figure 7. The thermal stability increases even more by elevating the percentage of the nanoparticles from SNP1 to SNP2. The weight loss in the glycerol-rich phase stage is a little bit less for the SNP films with greater nanoparticle content that illustrates fewer glycerol chains.

### 3.7 Water Contact Angle of SNP Films

**Figure 8:**
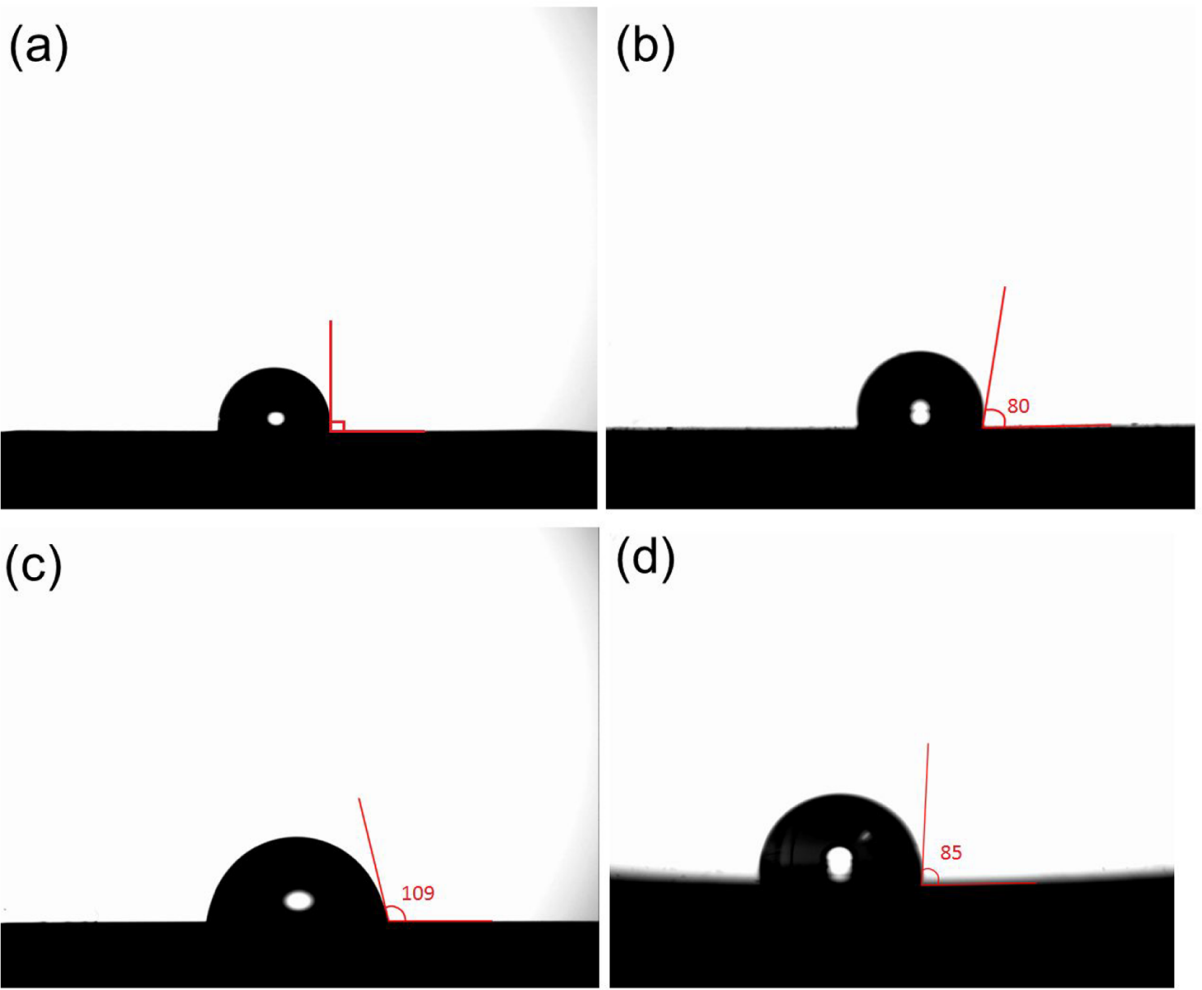
Contact angle images of SNP1 (a), SNP2 (b), SNP3 (c), and SNP4 (d).

The method which normally has been used to compare the hydrophobicity of starch film surfaces is the determination of the contact angle (CA) of water droplets on the surfaces.^31^ A high contact angle indicates better hydrophobicity of the surface and vice versa. The CA value of SNP films is presented in Figure 8. In the images captured, it is evident that the SNP films have significantly higher CA values than the starch-only films. According to these results, the SNP films exhibit greater hydrophobicity than the starch-only ones.^32,33^ Additionally, the figure shows that increasing the gelatinization time and glycerol content of the films results in a greater contact angle (the CA value are 90, 100, 71, and 85 responding to SNP1, SNP2, SNP3, and SNP4, respectively). These results suggest that there is a strong interaction between starch and nanoparticles.

**Figure 9:**
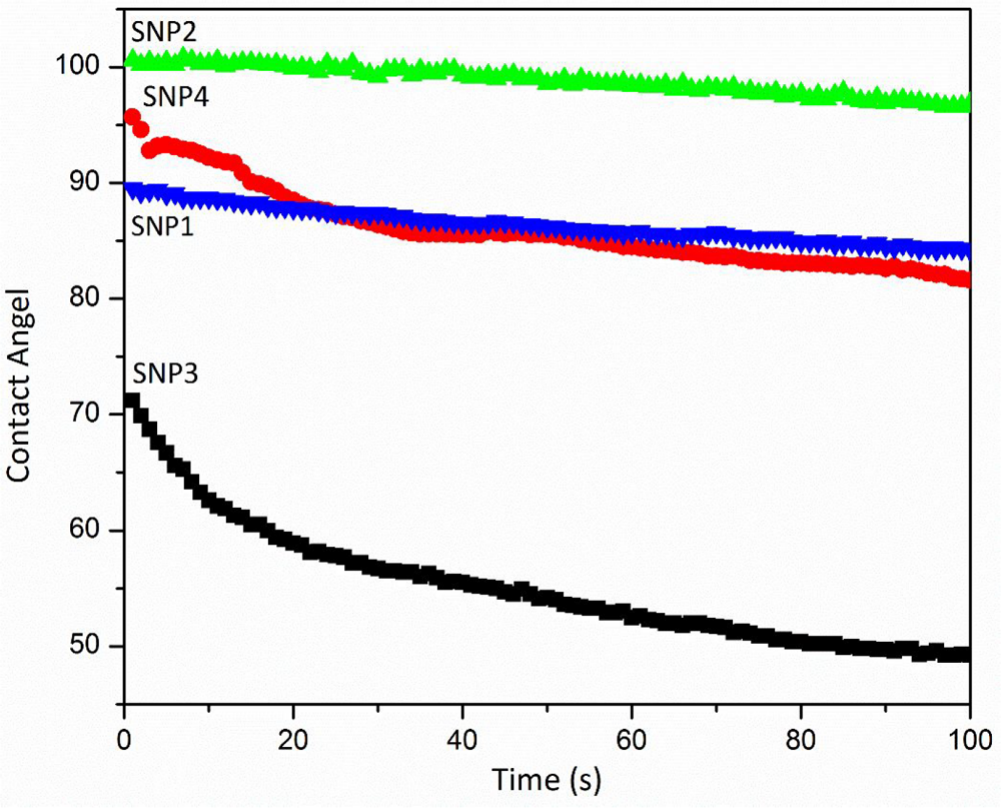
Contact angle measurements of all prepared SNP films over time.

Figure 9 demonstrates that the contact angle of SNP3 decreases with the time, and it means that the water droplet is absorbing to the surface of the film. However, for SNP1, SNP2, and SNP4, the reduction rate of contact angle decreased; it means that an increase in gelatinization time and glycerol content leads to stabilization of the water droplet on the surface of the mentioned film. So, the rate of the water absorption on the surface of the SNP film decreased compared to neat starch.

## 4 Conclusion

We have successfully produced SNP-containing starch films with hot and cold gelatinized starch solutions retaining ethanol in a single step. As a result of adding starch nanoparticles to the starch film, its wettability is reduced, and the existence of the plasticizer resulted in more homogeneous and more hydrophobic film. Furthermore, all samples containing SNPs displayed improved mechanical properties, which is confirmed by DMA results. Compared with starch films that lack glycerol, nanocomposites with SNPs and glycerol show more flexibility. According to the results of FTIR, the accretion in the intensity of the peaks by the addition of glycerol is perceived veritably, which is associated with the glycerol and SNP interactions and the matrix of starch. The homogeneous dispersion of SNPs and their interaction with the starch nanocomposites are confirmed by SEM. On the other hand, AFM shows the exact topography of distributed SNPs and different zones enriched with starch and SNPs after the nanoprecipitation process. XRD patterns demonstrate reduction crystallinity compared to neat starch granules, and it is changed from A-style to V_H_-style after precipitation. Moreover, thermal stability of nanocomposites is improved by adding the nanoparticles into the nanocomposites. Finally, surface hydrophobicity is increased on the samples by adding SNPs and leads to more stabilization compared to the samples without the nanoparticles.
